# A Method for Diagnosing Gearboxes of Means of Transport Using Multi-Stage Filtering and Entropy

**DOI:** 10.3390/e21050441

**Published:** 2019-04-27

**Authors:** Tomasz Figlus

**Affiliations:** Faculty of Transport, The Silesian University of Technology, 8 Krasinskiego Street, 40-019 Katowice, Poland; tomasz.figlus@polsl.pl; Tel.: +48-32-603-41-46

**Keywords:** condition monitoring, digital filtering, Walsh–Hadamard transform, entropy, gearbox

## Abstract

The paper presents a method of processing vibration signals which was designed to detect damage to wheels of gearboxes for means of transport. This method uses entropy calculation, and multi-stage filtering calculated by means of digital filters and the Walsh–Hadamard transform to process signals. The presented method enables the extraction of vibration symptoms, which are symptoms of gear damage, from a complex vibration signal of a gearbox. The combination of multi-stage filtering and entropy enables the elimination of fast-changing vibration impulses, which interfere with the damage diagnosis process, and make it possible to obtain a synthetic signal that provides information about the state of the gearing. The paper demonstrates the usefulness of the developed method in the diagnosis of a gearbox in which two types of gearing damage were simulated: tooth chipping and damage to the working surface of the teeth. The research shows that the application of the proposed method of vibration of signal processing enables observation of the qualitative and quantitative changes in the entropy signal after denoising, which are unambiguous symptoms of the diagnosed damage.

## 1. Introduction

Gearboxes are typical components of the propulsion systems of means of transport [1,2,3]. Their operation directly affects the reliability of the entire propulsion system [4,5]. Vibration signals of gearboxes of propulsion systems contain, first of all, characteristic information about the operation of the gears and the shaft bearings, and the main reason for the occurrence of vibrations are changes in the stiffness of the interacting elements, as well as changes in their technical condition. These signals are also modulated by signals containing information about the interaction and changes in the technical condition of other elements of the propulsion system [6,7,8,9,10,11,12,13,14]. This makes the vibration signal of gearboxes highly complex and often difficult to interpret. An important factor hindering the interpretation of the components of the vibration signals of gearboxes is the significant energy diversity of their components [15,16,17]. This prompts the search for new signal processing methods, which can enable quick diagnosis of the vibration condition of gearboxes based on their recorded vibration signal. 

Overloads, material defects, or contamination of the components of the means of transport can cause accelerated wear or damage to gearboxes. Wear or damage to the gearing of the wheels and shaft bearings can potentially occur most frequently. Pitting, chipping, or breakage of the teeth may occur in wheel gearing. Research on the detection of this type of damage is presented, inter alia, in [4,15,18,19,20,21,22,23,24,25,26,27,28,29,30,31,32,33,34,35,36,37,38,39,40,41,42]. In the case of bearings, pitting on the bearing elements or cracks in the race or balls can be encountered. Research in this area was carried out, among others, by authors of the papers [15,43,44,45,46,47,48,49,50,51,52,53,54,55,56,57,58,59,60]. It is possible that the above-mentioned changes in the condition of the elements of gearboxes will not cause any symptoms which are easily detectable in their initial stages by the users or service technicians of the means of transport. However, the growing damage can cause significant costs associated with, e.g., unplanned stopping of the means of transport or more extensive breakdowns. 

Various methods for the diagnosis of the rotating parts of means of transport, including gearboxes, have been in development for years. The most popular methods for diagnosis include recording vibration signals and then processing them in search of symptoms of a change in the technical condition. Contact methods, e.g., with piezoelectric transducers [4,5,7,14,15,24,25,26,27,28,29,35,45,47,56], and non-contact methods with laser vibrometers [7,14,15,36] are used for recording. The latter are used for measurements in hard-to-reach places and enable measurements of rotating shafts. Various methods of processing signals recorded in this manner are under development. Papers [5,29,34,39,43,47,51,60,61,62,63] suggested calculations of measures such as kurtosis and the skewness factor to diagnose the condition of gearboxes. A number of papers [36,47,51,55,60] propose the use of synchronous averaging and filtering. The use of time-frequency methods for detecting gearbox damage—results inter alia in [17,21,24,26,33,36,37]—makes it possible to determine the location of the damage characterised by fast-changing and short-lived vibration impulses. An additional advantage of the time-frequency methods is the ability to observe the change of the vibration signal in the selected frequency bands—Wigner–Ville’s time-frequency distribution [15], or the direct possibility of filtering—a discreet and continuous wavelet transform [21,22,26,33,37].

The paper presents a new method for processing the vibration signal of a gearbox in means of transport, which enables quick detection of damage to gearing. The method combines multi-stage filtering and calculation of the entropy of the vibration signal. The presented method enables the extraction of vibration symptoms, which are symptoms of gear damage, from a complex vibration signal of a gearbox in means of transport. The use of multi-stage filtering and entropy in the method enables the elimination of fast-changing vibration impulses, which interfere with the damage diagnosis process.

## 2. Signal Processing Method

In this paper, a method of multi-stage filtering of the vibration signal and entropy calculation, dedicated to the detection of damage to the gearing of gearboxes in means of transport, was developed. 

In the spectrum of the vibration signal of gearboxes, high-energy and low-energy components can be distinguished. The high-energy components of such a signal are contained in the first few rotational harmonic frequencies of the pinion and the gear and in the subsequent harmonic frequencies of the gearing of the wheels together with sidebands. A low-energy signal is a signal between the high-energy components of the vibration spectrum. The use of digital filtering—discussed in detail hereinafter—for signal processing enables the extraction of the low-energy components of vibration signals, which contain information about gearing damage, from a complex high-energy signal of the gearbox. In order to clearly separate the low-energy and high-energy components of the vibration signal, as well as to identify the individual types of damage, digital filters should be characterised by steep slopes of amplitude-frequency characteristics and the lack of phase shifts and uncontrolled amplitude excitation. The calculation of entropy in the walking window of a low-energy signal enables an unambiguous indication of the qualitative and quantitative changes caused by the damage. The method also proposes the use of filtering with a Walsh–Hadamard transform. This approach allows additional denoising of the entropy signal, and the obtained signal becomes more legible and clearer in the diagnosis process. 

At the beginning of the development of the signal processing method, it was assumed that the computer program created in the Matlab environment would work quickly and automatically and would require only the input of basic data and calculation assumptions.

**Figure 1:**
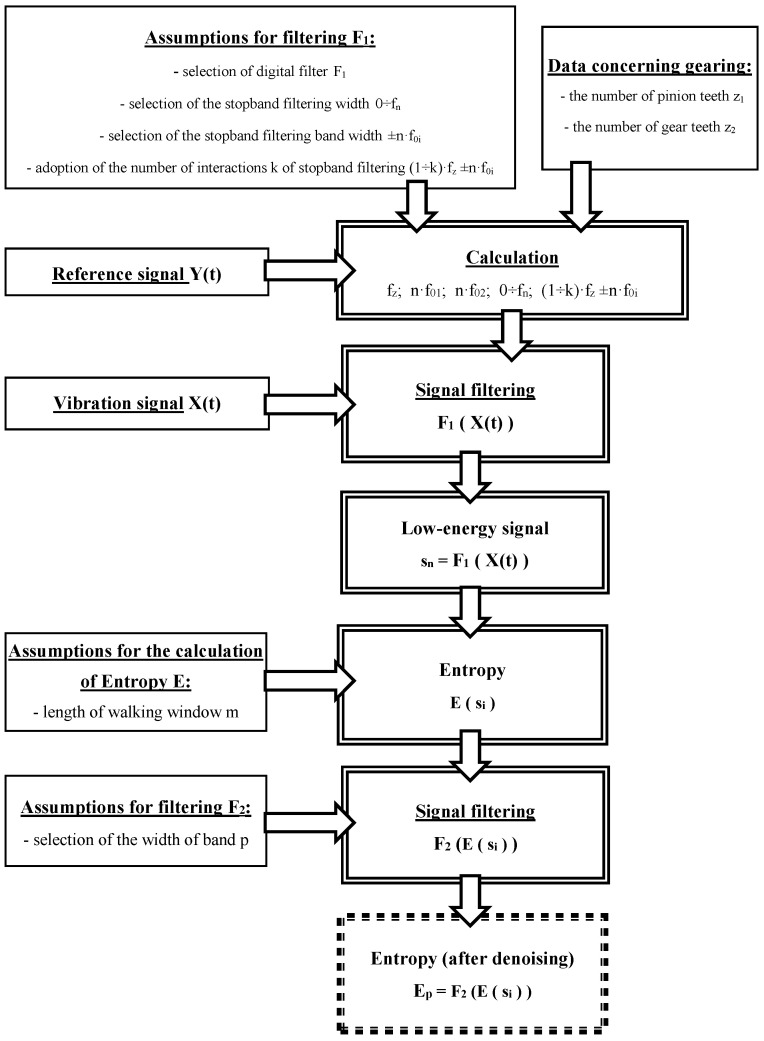
The procedure for signal processing.

Figure 1 shows an algorithm developed for signal processing in the proposed method. The following data must be provided before signals can be processed:

1. Assumptions for filtering F_1_, i.e.,
  - selection of digital filter F_1_
  - selection of the stopband filtering width 0–f_n_
  - selection of the stopband filtering bandwidth ±n·f_0i_
  - adoption of the number of interactions k of stopband filtering (1–k)·f_z_ ± n·f_0i_
2. Data concerning gearing:
  - the number of pinion teeth z_1_
  - the number of gear teeth z_2_
3. Reference signal Y(t)—it enables the calculation of rotational speed (n_o_), as well as the values in items 1 and 2
4. Assumptions for the calculation of Entropy E:
  - length of walking window m
5. Assumptions for filtering F_2:_
  - selection of the width of band p

Next, the recorded vibration signal X(t) is transmitted for processing and calculations are carried out.

**Figure 2:**
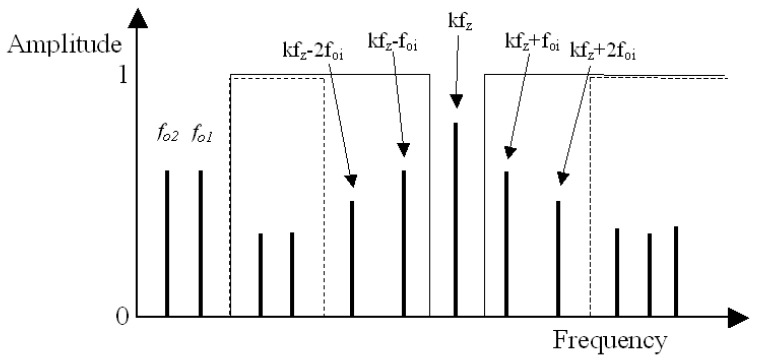
Design of the residual and difference filter: solid line presents the bandpass of the residual filter; the dashed line presents the bandpass of the difference filter; f_oi_ represents the rotation of the wheels, and k represents the harmonics 1,2,3.

The method assumes that the signal containing the information about the condition of the gearing will be filtered out of the recorded vibration signal of the gearbox by a multi-stage digital differential filter with very steep amplitude-frequency characteristics (Figure 1), designated F_1_. This will enable the separation of the low-energy components from the high-energy components of the analysed signal. In the first interaction of the filtering process, high-energy frequency components in the 0–f_n_ range, which contain the rotational frequencies of pinion f_02_ and gear f_01_ of the gearbox, and their subsequent harmonics, are filtered out of the signal. Then, in subsequent interactions, the gear mesh frequency f_z_ and their harmonics k·f_z_, along with the sidebands ±n·f_01_ and ±n·f_02_, are filtered out of the signal. Based on the filtering carried out, low-energy signal s_n_ will remain for further research. Similar assumptions for the configuration of the filtering bands (Figure 2) were made, among others, in the research presented in papers [15,16,17,64].

Based on the low-energy signal s_i_ obtained after filtering, the entropy is calculated in a walking window with length m based on of the following dependency:

(1) $$E\left(s_{i}\right)=-\sum_{n=i}^{i+m}s_{n}^{2}\log\left(s_{n}^{2}\right)$$

where *s_i_*—low-energy signal, *m*—length of the walking window.

The author’s research shows that frequently in vibroacoustic signals, after the application of the processing of signals that have not been averaged synchronously, components are present that continue to interfere with the diagnosis process. Therefore, the developed method proposes an additional filtering F_2_, carried out with the use of a fast Walsh–Hadamard Transform. Based on the author’s research and the results presented in [65,66,67,68], it can be concluded that a fast Walsh–Hadamard Transform can be very useful for reducing non-correlated components of the signals, which constitute signal noise.

## 3. Test Stand 

A number of laboratory experiments were carried out in order to obtain a wide range of vibration signals emitted by an actual gearbox in which various types of damage occurred. During active experiments, vibration signals of the gearbox, in which various types of gearing damage were simulated, were recorded. 

**Figure 3:**
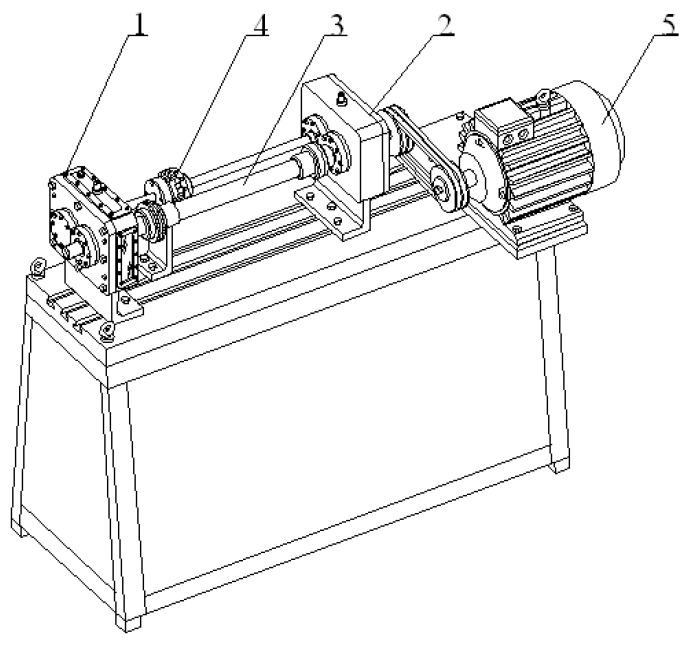
Circulating power test bed FZG. It consists of the examined gear (1), closing gear (2), torsion bar (3), tightening clutch (4) and an electric motor (5).

The measurements were carried out in the Power Transmission Systems Laboratory of the Faculty of Transport of the Silesian University of Technology in Katowice (Poland), on the circulating power test bed FZG (Figure 3). This stand enables the testing of gearboxes, among others for damage to the gears of wheels [34,36]. 

An object of investigation were gear wheels with the following parameters presented in Table 1.

**Figure 4:**
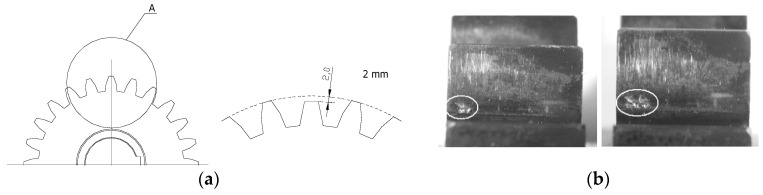
Simulation of the tooth chipping (**a**) and wear on the surface of the teeth (**b**).

In the first experiment, 2 mm tooth chipping was simulated (Figure 4a). It was caused by the decrease in the value of the contact ratio from 1.32 to 0.93. In the second experiment, the wear of the working surface of the teeth was simulated (Figure 4b). In these experiments, it was found that the wear on the surface of the teeth of the wheels was unevenly distributed across the individual teeth [34,36]. 

**Figure 5:**
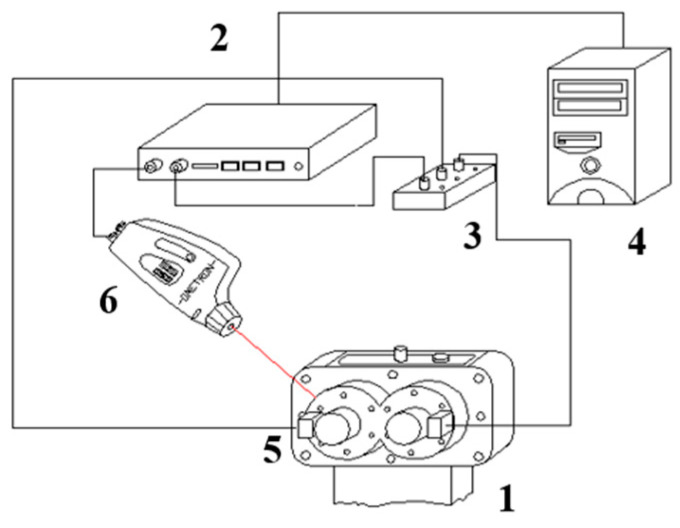
Measuring system diagram consists of examined gear (1), DSPT SigLab signal analyser (2), logic unit (3), PC (4), shaft angle position sensors (5), and laser vibrometer (6).

During the test, an Ometron laser vibrometer was used to measure the speed of the transverse vibration of the shafts. Furthermore, additional impulses corresponding to the consecutive periods of the pinion and gear teeth meshing were recorded (Figure 5). The sampling frequency was established at 25.6 kHz.

The experiment was carried out with a significant unit load on the gears, amounting to Q = 3.85 MPa, which was set on the stand by means of a tensioning clutch. The rotational speed of the gear shaft was 1800 rpm.

## 4. Results and Discussion

A calculation program was developed in Matlab based on the signal processing method proposed in Section 2. The method and the program were tested using signals recorded in accordance with the information provided in Section 3. 

During the preliminary research, the usefulness of the developed method was analysed by testing the functioning of the program using recursive digital IIR filters and non-recursive digital FIR filters. This research shows that IIR filters make it possible to obtain a steep slope of amplitude-frequency characteristics of the filter and short time of the multi-band filtering. In order to eliminate phase disturbances—phase shifts—the digital filtering method with a delayed zero phase was used during filtering. This approach makes it possible to preserve the qualitative characteristics of changes in the filtered signal exactly where they occur in the signal before filtering.

The further part of the paper presents examples of the results of the wheel damage diagnosis process, which were obtained based on the following calculation assumptions:

1. Assumptions for filtering F_1_, i.e.,
  - selection of digital filter F_1_—recursive digital IIR filters with the delayed zero phase, stopband −65 dB
  - the stopband filtering width 0–100 Hz
  - the stopband filtering width ±100 Hz
  - the number of interactions k of the stopband filtering 6
2. Data concerning gearing:
  - the number of pinion teeth 16
  - the number of gear teeth 24
3. Reference signal Y(t)—rotational speed: 1800 rpm
4. Assumptions for the calculation of Entropy E:
  - length of walking window 10
5. Assumptions for filtering F_2:_
  - fast Walsh–Hadamard Transform
  - selection of the width of band 550

**Figure 6:**
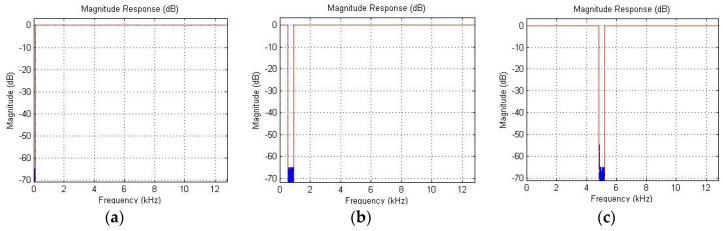
Examples of digital band filtering: band 0–100 Hz (**a**); band 1·720 ± 100 Hz (**b**); band 6·720 ± 100 Hz (**c**).

**Figure 7:**
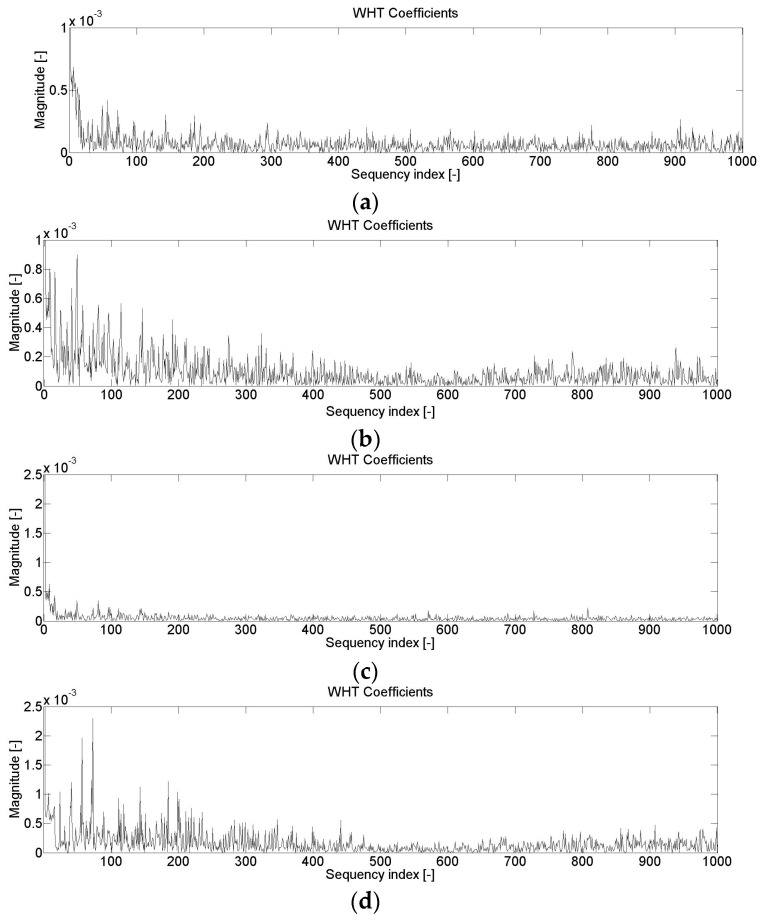
Changes in the values of the Walsh–Hadamard Transform vibration signal—diagnosis of tooth chipping: no damage (**a**) and 2 mm chipping (**b**); diagnosis wear of work surface of the teeth: no damage (**c**) and damage to the working surface of teeth (**d**).

Figure 6 shows three examples of digital band filtering, and Figure 7 shows examples of the effect of tooth chipping and damage to the working surface of teeth on the change of entropy distribution by means of a fast Walsh–Hadamard Transform. 

The Walsh-Hadamard entropy distributions shown in Figure 7 indicate that for both the diagnosis of chipping and the wear of the teeth’s working surface, the main changes in these distributions occur in the 0–550 range. Therefore, it was assumed for further research that denoised entropy would be determined for the above-mentioned range.

**Figure 8:**
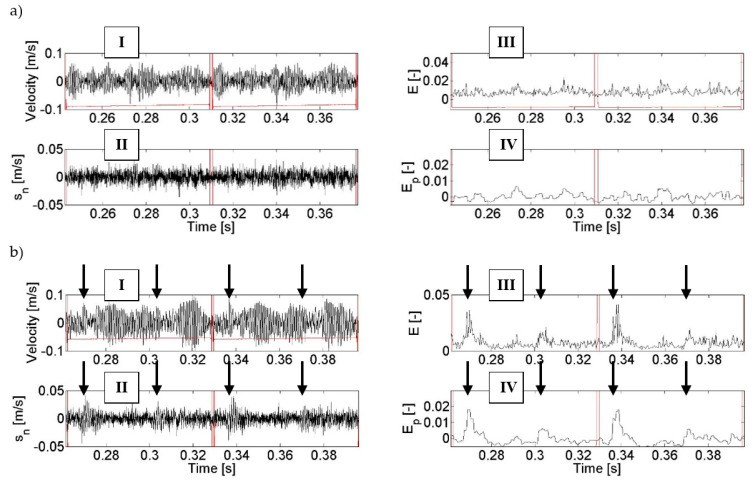
Changes of the values of the recorded and processed vibration signals during diagnosis of tooth chipping: no damage (**a**) and 2 mm chipping (**b**), where: I—raw vibration signal, II—low-energy signal, III—entropy of low-energy signal, and IV—entropy of low-energy signal after denoising.

**Figure 9:**
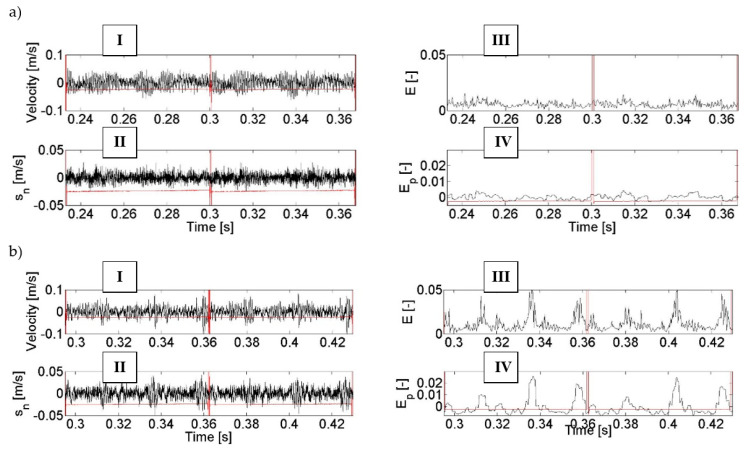
Changes of the values of the recorded and processed vibration signals during the diagnosis of the wear of the working surface of the teeth: no damage (**a**) and damage to the working surface of teeth (**b**), where: I—raw vibration signal, II—low-energy signal, III—entropy of low-energy signal, and IV—entropy of low-energy signal after denoising.

The following Figure 8 and Figure 9 show the results of the vibration signal analyses carried out in order to detect the gearing damage discussed in Section 3: tooth chipping and damage to the working surface of teeth. These figures show selected graphs with the changes in signals over time for two subsequent cycles of gearing couples. 

The results of this research indicate that the use of the F_1_ filter enabled the filtering of low-energy components (designated II) out of a high-energy vibration signal (designated I). Entropy E (designated III) determined in further calculations clearly shows the amplitude changes which are symptoms of tooth damage (Figure 8b and Figure 9b) as compared to the condition of an intact gearbox (Figure 8a and Figure 9a). Additional filtering F_2_ of the entropy signal E resulted in the removal of the interfering components from this signal and thus in better highlighting of the symptoms of the damage in the entropy signal E_p_ (designated IV in Figure 8b and Figure 9b).

An analysis of the calculation results obtained in the process of diagnosis of the chipping of a tooth tip led to the conclusion that filtering and calculation of entropy made it possible to obtain an unambiguous energy symptom in the form of a locally increased signal amplitude (marked with arrows in the figures). This increase is observed in the range of the angle of rotation of the wheel in whose tooth contact there is a damaged tooth of the gear and the teeth interacting directly after it. It can be observed that for gearboxes without damage to the gearing, the trend of entropy change after denoising E_p_ is in the range of −0.003–0.008 and it is not possible to unambiguously find in this signal periodically repeated local amplitude increases—in accordance with the rotation of the pinion or the gear. In the case of the presence of the modelled tooth chipping in the signal, a significant local increase in entropy amplitude of up to E_p_ = 0.018 and 0.005 is observed, depending on the cycle of the interactions of the teeth—marked with arrows in Figure 8. The differences in values of the amplitude increase are caused by the fact that the analysed signal has not been averaged synchronously, and therefore there are non-linearities related to, among others, the dynamics of the analysed power transmission system or errors made in the production of the gearing and other elements.

When looking for signs of wear on the working surface of teeth, it can be noticed that the application of the signal processing method proposed in the paper also influenced the possibility of observing qualitative and quantitative changes in signals caused by wear. When comparing entropy signals after denoising E_p_, it can be seen that the trend in the signal amplitude change when the gears were in a good condition is similar to that of the pair of wheels tested in the previous experiment. On the other hand, when there is damage to the working surface of the pinion teeth, a threefold increase in entropy amplitude can be clearly observed (which results from the cycle of interactions of the tested pair of wheels), which is a clear symptom of damage to the pinion. Maximum local values of entropy, in this case, are 0.011; 0.023, and 0.02, respectively. The range of changes in entropy amplitude indicates that the damage occurs on more than one pinion tooth. The course of these changes is of a different nature, as the wear did not appear on all the teeth of the pinion proportionally, but to varying extents. Similarly to what was observed in the analyses of a signal containing symptoms of tooth chipping, also in this case the lack of averaging, the dynamics of the propulsion system, and the errors in production cause the amplitude of the signal in subsequent association cycles to change in an undetermined manner. 

Analysing the results obtained in the study, it can also be concluded that if the value of entropy after denoising is Ep > 0.01, for the gear under examination, a change in the condition of the toothed wheels occurs due to their damage or wear. Where a gear is tested in normal operation, the results of such calculations should refer to past measurements of the gear, when it was in good working order and operated under similar conditions. A comparison of the entropy value after denoising Ep for the initial and current states of the gear will enable an unambiguous assessment of its technical condition.

## 5. Conclusions

The paper presents a new method of processing vibration signals, in which multi-stage filtering and calculation of entropy were used. The computer program developed in a Matlab environment enables fast signal processing and makes it possible to obtain unambiguously informative signal synthetics. This solution can, therefore, be used to compare multiple mechanical systems with each other in a short time based on single samples of time signals of a certain length.

The presented signal processing method is aimed at diagnosing damage to gears of gearboxes in propulsion systems. This approach to signal processing made it possible, in the case under consideration, to distinguish low-energy symptoms of damage and wear of wheels from a high-energy vibration signal. 

Therefore, the approach of signal processing presented in the paper can become a fast computational tool for diagnosing damage and wear of wheels of gearboxes in propulsion systems. 

## Figures and Tables

**Table 1:** Gear wheel parameters.

Number of pinion teeth	16	**-**
Number of gear teeth	24	-
Face width	20	mm
Normal module	4.5	mm
Coefficient of pinion addendum modification	0.864	-
Coefficient of gear addendum modification	−0.5	-
Distance between the centres of two gears	91.5	mm
Helix angle	0	°
Hardness (diagnosis of the chipping of a tooth tip)	60–62	HRC
Hardness (diagnosis of the wear of the teeth’s working surface)	37–40	HRC
